# Ultra-Low Frequency TENS as an Adjunctive Therapy for Pain Management in Non-Surgical Periodontal Treatment: A Pilot Study

**DOI:** 10.3390/dj13040161

**Published:** 2025-04-09

**Authors:** Eleonora Ortu, Sara Di Nicolantonio, Roberta Di Felice, Antonella Barone, Davide Pietropaoli, Annalisa Monaco

**Affiliations:** Division of TMD and Orofacial Pain, Department of Clinical Medicine, Public Health, Life Sciences and the Environment, University of L’Aquila, 67100 L’Aquila, Italy

**Keywords:** ULF-TENS, pain relief, non-surgical periodontal therapy, scaling, root planing, discomfort reduction

## Abstract

**Introduction:** non-surgical periodontal treatment, primarily comprising scaling and root planing, is crucial for the maintenance and enhancement of oral health. However, the invasive nature of this procedure often leads to patient discomfort and pain, which may deter individuals from seeking necessary dental care, ultimately compromising their oral health outcomes. **Methods:** This prospective randomized crossover split-mouth study involved the application of Ultra-Low Frequency (ULF) Transcutaneous Electrical Nerve Stimulation (TENS) in 20 adult patients undergoing non-surgical periodontal treatment. Pain and discomfort levels were quantitatively assessed during procedures conducted with and without the ULF-TENS intervention. **Results:** The assessment of maximum voluntary opening, pain intensity, and overall comfort levels indicated a statistically significant reduction in pain (*p* < 0.0001) and discomfort (*p* < 0.0001) when ULF-TENS was employed during the treatment, and an increase in the maximum mouth opening after TENS (*p* = 0.00062). **Conclusions:** The findings of this pilot study suggest that ULF-TENS may serve as a valuable adjunctive therapy in non-surgical periodontal treatment by reducing pain and discomfort, potentially enhancing patient comfort and compliance. Further research with larger sample sizes is warranted to confirm these findings.

## 1. Introduction

Non-surgical periodontal treatment (NSPT), which encompasses scaling and root planing, is recognized as the preferred intervention for maintaining oral health in both periodontitis-affected and healthy patients. This treatment involves the application of various manual tools (such as curettes and scalers) and ultrasonic devices designed to eliminate hard and soft deposits from both supra- and subgingival areas, thereby fostering an oral environment conducive to periodontal health [1,2]. While NSPT is critical for preventing periodontitis and associated diseases, it can often be uncomfortable and painful for patients. Research by Graziani et al. [3] indicates that intraoperative pain is one of the most prevalent complications of NSPT, influenced by the type of instruments used, the characteristics of their tips, and the individual pain tolerance of each patient. Additionally, ongoing inflammatory processes in the gingival tissue may exacerbate pain sensitivity, further lowering the pain threshold [4].

Such discomfort can significantly impact patient compliance, leading individuals to forgo routine dental hygiene appointments to avoid painful experiences. Consequently, patients may only seek dental care after the onset of more severe conditions, which can result in higher costs, increased time in the dental chair, and potentially greater pain during treatment [5]. Pain perception is inherently subjective and influenced by various factors, including systemic and psychological components such as fear, anxiety, and stress. To assess these dimensions, several indices are employed, including the DASS21 for anxiety and stress, and the Visual Analogue Scale (VAS) for pain measurement [6,7]. Notably, a study using the VAS indicated that approximately 52.3% of patients experienced pain or required analgesics within two hours post-scaling and root planing under local anesthesia [8,9]. The literature reveals that the percentage of patients who report no pain during the procedure varies widely, from 10% [10] to 36% [9].

Current pain management strategies for NSPT predominantly involve pharmacological approaches using local anesthetics. However, the array of available medications and treatment protocols can complicate clinical decision-making in routine practice [11]. Furthermore, the presence of concurrent health issues such as hypertension, cardiovascular conditions, and metabolic disorders necessitates careful consideration in the selection and application of local anesthetics to mitigate potential side effects [12,13].

Given these challenges, the adoption of innovative techniques and technologies for pain management is essential. Ultra-Low Frequency TENS (Transcutaneous Electrical Nerve Stimulation), particularly applied at the tragus, has been utilized in dentistry for chronic pain management in patients with temporomandibular disorders (TMDs) [14]. This technique aims to stimulate neural structures, specifically the trigeminal and facial nerves, providing impulses to the stomatognathic muscles that may exhibit reduced tone. Research indicates that ULF-TENS can yield not only neuromuscular benefits but also significant humoral and analgesic effects [15]. It is believed to activate the periaqueductal gray (PAG) and rostroventral medulla (RVM) pathways in the spinal cord, promoting the release of endorphins, which play a crucial role in pain modulation [16].

The current hypothesis posits that ULF-TENS can diminish pain and the production of catabolic substances by inhibiting the activity of the Locus Coeruleus, a key producer of norepinephrine and a central regulator of alertness, stress, and pain perception during dental visits [17].

This study aims to evaluate the effectiveness of ULF-TENS in managing pain during NSPT, utilizing both the VAS and DASS21 scales, as well as measuring maximum mouth opening (MVO) in 20 healthy patients. We hypothesize that the application of ULF-TENS during non-surgical periodontal treatment may have a positive effect on pain perception, mouth opening, and patient comfort. Consequently, it could serve as an adjunctive treatment for pain management during dental procedures.

## 2. Materials and Methods

This study was conducted in accordance with the fundamental principles of the Declaration of Helsinki and was approved by the Ethics Committee of the University of L’Aquila (Approval Number: 16137/2016). This study was registered on clinicaltrials.gov (ID: NCT06870747).

### 2.1. Patient Selection

- A total of 45 patients from the Preventive Service of the Dental Clinic of the University of L’Aquila, seeking non-surgical periodontal therapy, were initially enrolled based on the following inclusion and exclusion criteria:

### 2.2. Inclusion Criteria

Healthy patients aged 30 to 75 years, of both sexes, seeking non-surgical periodontal treatment.

### 2.3. Exclusion Criteria

- Patients undergoing opioid or analgesic therapies in the last month.
- Pregnant women.
- Patients with a history of epilepsy.
- Patients with implanted electronic devices (e.g., pacemakers, implantable cardioverter defibrillators).
- Patients with systemic or regional neuropathies (e.g., polyneuropathies, neuropathies associated with viral infections).
- Patients with chronic systemic degenerative diseases (e.g., multiple sclerosis, Parkinson’s disease, advanced diabetes with neuropathy).
- Patients with chronic pain conditions (e.g., fibromyalgia, chronic lower back pain, osteoarthritis, tendinitis, bursitis, chronic pelvic pain).
- Patients with pulpitis, pulpal diseases, temporomandibular disorders (TMDs), orofacial pain, periodontitis, or any other oral condition associated with oral pain.
- Patients who did not sign the informed consent form.

Of the 45 initially enrolled patients, 25 were excluded: 17 refused to participate, and 8 had recently undergone analgesic therapies. As a result, the final sample consisted of 20 patients (10 males and 10 females, aged 30–75 years). Although the initial inclusion criteria considered patients over 18 years old, those under 30 either withdrew from the study or did not provide final consent. All selected patients signed the informed consent form. Prior to study enrollment, all participants underwent a periodontal examination performed by a dental hygienist, which included the compilation of a periodontal clinical chart to confirm the absence of periodontitis or other conditions that could influence the study outcomes.

### 2.4. Study Design

**Figure 1:**
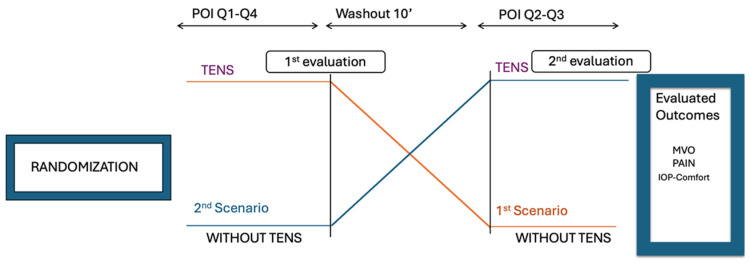
Organization of the study.

This study was a randomized, controlled, crossover split-mouth trial designed to evaluate the effects of Transcutaneous Electrical Nerve Stimulation (TENS) on pain, discomfort, and maximum voluntary mouth opening (MVO) during non-surgical periodontal therapy. The overall study design is illustrated in Figure 1.

Each participant underwent periodontal treatment under two experimental conditions:

1. Quadrant 1 (TENS treatment): 10 min non-surgical periodontal treatment with TENS activated.
2. Quadrant 4 (No TENS treatment): 10 min non-surgical periodontal treatment without TENS.

a. After a 10 min break:

3. Quadrant 2 (TENS treatment): 10 min non-surgical periodontal treatment with TENS activated.
4. Quadrant 3 (No TENS treatment): 10 min non-surgical periodontal treatment without TENS.

Post-treatment evaluations were conducted to compare the effects of TENS versus non-TENS treatment across different quadrants.

### 2.5. Randomization

Patients were randomly assigned to the two groups using dedicated software (https://www.sealedenvelope.com/, accessed on 19 Genuary 2024) following a 1:1 stratified allocation procedure with variable block randomization (block sizes of 4, 6, and 8). The randomization process was performed by a research assistant who was not involved in data collection or analysis.

### 2.6. Sample Size Determination

The sample size was calculated using G*Power software (3.1. 9.4), with an expected effect size of 0.66, a significance level (α) of 0.05, and a power of 0.80. Based on these parameters, an initial sample size of 16 participants was determined. However, to account for potential contingencies such as dropouts or missing data, a final sample size of 20 participants was selected. The use of a crossover split-mouth design provided additional statistical power by reducing inter-subject variability, allowing for a smaller sample size and increasing the precision of the findings [18,19]. A purposive sampling approach was employed to deliberately select participants according to predefined eligibility criteria, maximizing the relevance and applicability of the findings [20].

### 2.7. Clinical Procedure

After the submission of the informed consent, model Myomonitor TENS Unit (Myotronics-Noromed, Inc., Tukwila, WA, USA) with disposable electrodes (Myotrode SG Electrodes, Myotronics-Noromed, Inc., Tukwila, WA, USA) was used. This ultralow frequency neurostimulator generates a repetitive, synchronous, bilateral stimulus with a frequency of 0.66 Hz, an adjustable amplitude ranging from 0 to 24 mA, and a pulse duration of 500 μs.

For tragus stimulation, the two TENS electrodes were placed bilaterally over the cutaneous projection of the notch of the fifth cranial nerve, located between the coronoid and condylar processes, which was identified through manual palpation of the area anterior to the tragus. A third reference electrode was placed at the center of the back of the neck.

Once the electrodes were placed, the operator gradually increased the stimulation amplitude until the sensory threshold was reached. The sensory threshold was identified when the patient first began to perceive the stimulus in the pre-tragal region. Following this, the staff conducted an NSPT session lasting 20 min, as previously described: 10 min with the ULF-TENS activated and 10 min with it deactivated. Each patient completed a DASS21 questionnaire before and after each session. The MVO was measured using a kinesiograph, equipment that can reproduce jaw movement in three dimensions on the computer.

To minimize measurement variability, all assessments were conducted by the same trained examiner following a standardized protocol. The placement of the electrodes was performed by an experienced operator specialized in the use of TENS for the diagnosis and treatment of temporomandibular disorders, ensuring consistency in stimulation. While intra-assessor agreement was not formally measured, the use of a consistent methodology aimed to ensure reproducibility of the results.

### 2.8. Statistical Analysis

Statistical analysis was conducted by a researcher unaware of the study procedures and rationale. The normality of the data was assessed using the Shapiro–Wilk test, which confirmed that all measurements followed a normal distribution. Paired-samples *t*-tests were used to compare the MVO, pain, and intensity before and after TENS and with and without TENS during NSPT. Additionally, to assess potential age-related variations, patients were categorized into three distinct age groups: 30–44, 45–59, and 60–75 years. ANOVA was performed for each variable (pain, comfort, and MVO) to examine differences across these age groups. Paired *t*-tests were also conducted within each age group to compare pre- vs. post-treatment conditions and with vs. without TENS. The significance threshold for the *p*-value was set at 0.05. All statistical analyses and visualizations were generated using R software version 4.0.2.

## 3. Results

**Figure 2:**
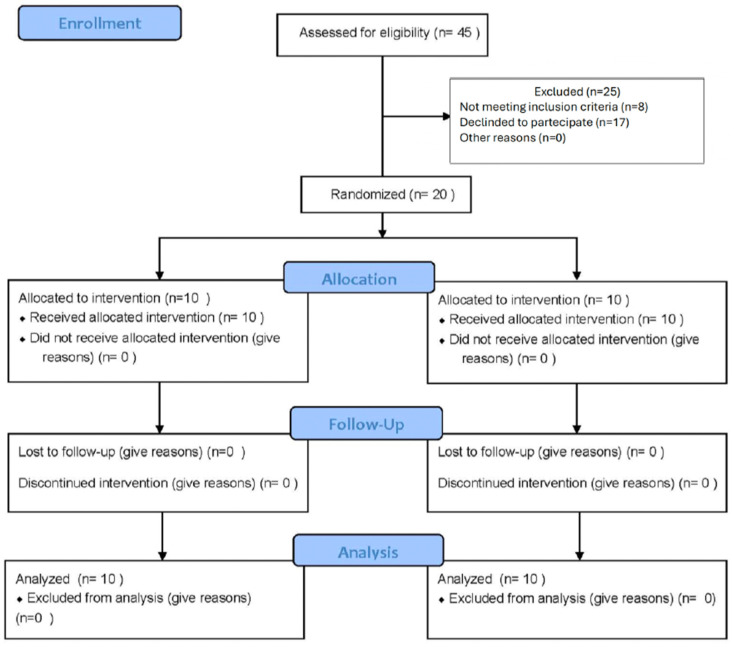
Flow diagram of study population’s enrollment and randomization.

In Figure 2 and Table 1 is reported the number of individuals at each stage of the study and the demographic characteristics of the two groups at baseline. For each patient, the authors compiled the collected data about personal information, pain and discomfort with and without TENS, D, A, and S after treatment, and MVO (maximum voluntary opening) before and after TENS. All results were tabulated. The aim, as previously illustrated, was to evaluate the effect on self-perception of discomfort, pain, and the possibility to open the jaw during and after the treatments the authors performed in this study.

**Figure 3:**
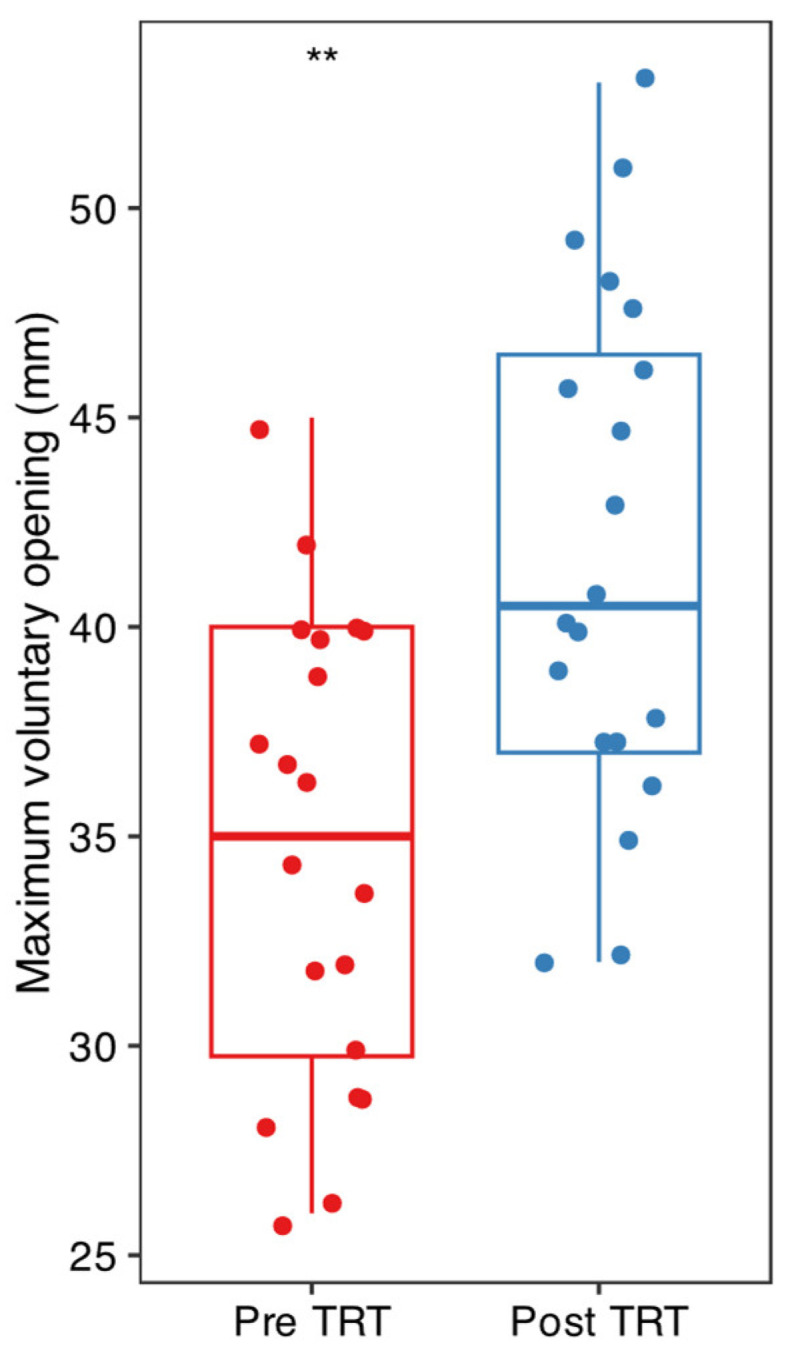
Paired *t*-test of MVO (maximum voluntary opening of the mouth) or MAV measured in millimeters with a gauge before and after TENS; ** *p* < 0.01.

**Figure 4:**
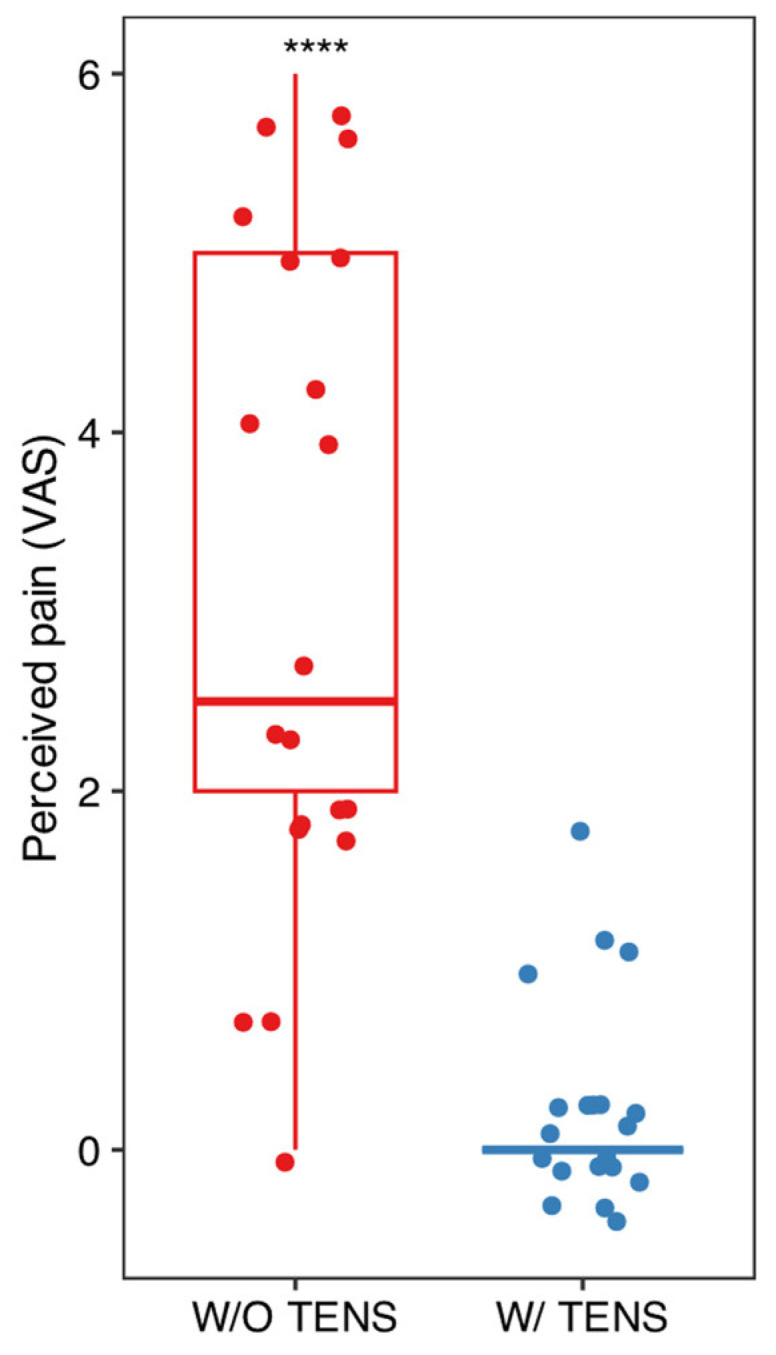
Comparison of pain levels with and without TENS using the VAS scale. Paired *t*-test was performed to assess statistical differences; **** *p* < 0.001.

**Figure 5:**
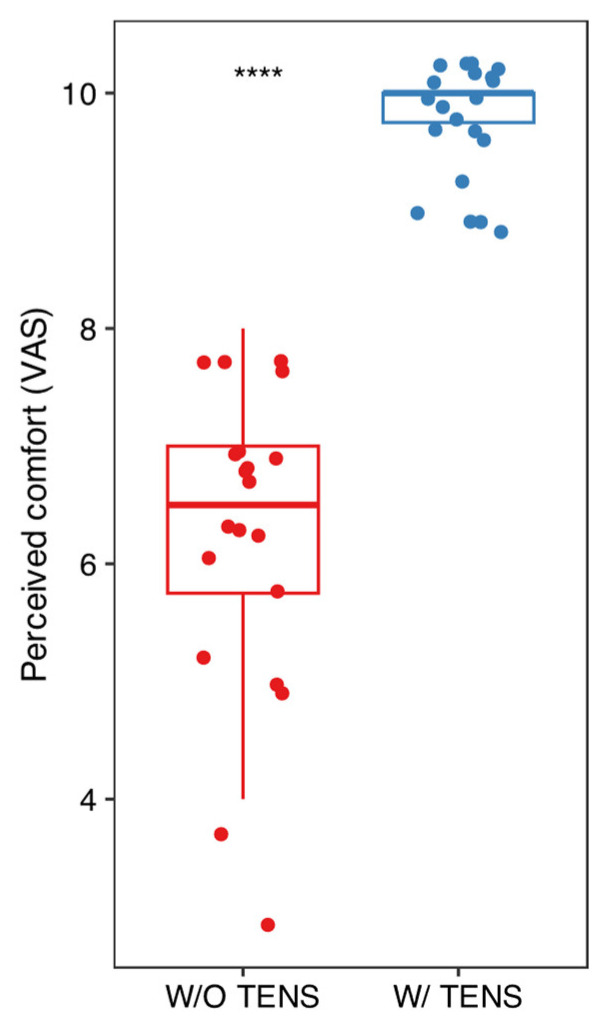
Comparison of comfort levels with and without TENS using the VAS scale. Paired *t*-test was performed to assess statistical differences; **** *p* < 0.001.

Mean and standard deviation of the collected data are listed in Figure 2, Figure 3 and Figure 4. Results were analyzed by the authors and a *t*-test statistical analysis was performed to determine whether there were statistically significant differences in the following variables [21,22,23,24]:

- MVO (maximum voluntary opening) before and after TENS (Figure 3);
- Pain intensity with and without TENS (Figure 4);
- Pain and discomfort with and without TENS (Figure 5).

This protocol was elaborated in order to limit the potential differences between two groups of distinct patients, randomizing the succession of TENS on and off. The authors also considered the low number of patients enrolled updating the randomization process.

T-test analysis for the three variables—MVO, pain intensity, and pain/discomfort—revealed statistically significant differences (before/after and with/without) when ULF-TENS was used in conjunction with NSPT. However, no statistically significant differences were observed across the age groups in any of the conditions (before/after and with/without TENS). Nevertheless, when the patients were considered within each age group, each parameter became statistically significant. This suggests that the effect of ULF-TENS on the measured outcomes is consistent across age groups, indicating an overall homogeneous response to the stimulation, regardless of age (see Supplementary Files for figures and graphs).

## 4. Discussion

The results of our study support the hypothesis that the use of ULF-TENS during non-surgical periodontal therapy (NSPT) can significantly improve patient comfort by reducing pain compared to treatment without the aid of this technology. “Dentist fear and anxiety” (FDA) is a well-known phenomenon that originates from negative past experiences and has a direct impact on the patient’s oral health. If not addressed, this condition can hinder access to dental care, compromising the quality of treatment. According to meta-analytic studies, fear of pain and dental anxiety affect approximately 15.3% of the global population, with higher prevalence in women and adults compared to younger individuals. These findings highlight the need for effective strategies for pain and anxiety management in dentistry [25].

The advent of new techniques for conscious sedation and relaxation has been widely welcomed in clinical practice for managing these situations. Midazolam and ketamine were among the first sedative medications introduced, offering hypnotic, analgesic, and muscle relaxant effects. However, their side effects were not negligible and required the presence of experienced staff to manage them [26].

Inhalation sedation with nitrous oxide, for pain, anxiety, and stress management, has been reported as an effective and very safe sedation method. It is commonly used in pediatric patients, though its use in adults is also well documented [27,28].

In this context, Transcutaneous Electrical Nerve Stimulation (TENS), particularly Ultra-Low Frequency TENS (ULF-TENS), emerges as a potential therapeutic resource for pain control and improving patient comfort during dental procedures. To the best of our knowledge, this is the first study experimenting with the use of ULF-TENS in non-surgical periodontal therapy. The application of this technique has yielded surprising and encouraging results, despite a small sample size, reducing the pain and discomfort frequently reported by patients during NSPT sessions.

Ultra-Low Frequency ULF-TENS was originally used for managing orofacial pain and diagnosing and treating temporomandibular disorders (TMDs)and now is also use in orthodontics [29]. In 2021, the National Institute for Health and Care Excellence (NICE), in its guidelines for managing chronic pain, recommended TENS as an adjunct in the treatment of osteoarthritis and rheumatoid arthritis [30,31]. Moreover, a meta-analysis of 381 articles on the effectiveness of TENS in pain management provides moderate-certainty evidence that it can clinically and significantly reduce pain intensity during and immediately after its use. This has led healthcare providers to consider TENS as an addition to baseline treatment, regardless of the specific diagnosis [32].

Numerous studies have demonstrated the effectiveness of Transcutaneous Electrical Nerve Stimulation (TENS) in modulating pain not only in temporomandibular disorders (TMDs) but also in other oral and dental pain conditions, highlighting its role as a non-pharmacological alternative to local anesthetics. Malamed et al. [33] evaluated electronic dental anesthesia (EDA), a modified version of TENS for intraoral use, showing that 89% of patients reported adequate pain control during restorative dental procedures without the need for additional local anesthesia. Moreover, one of the main advantages of using TENS is the complete absence of residual post-operative anesthesia, ensuring a rapid recovery without the numbness of soft tissues.

Roth and Thrash [33] further confirmed the effectiveness of TENS in reducing pain associated with orthodontic movements, demonstrating that patients treated with TENS reported a significant reduction in pain, assessed using the VAS scale, compared to control and placebo groups, up to 48 h after treatment. These results suggest that TENS can provide prolonged analgesic effects, making it a potentially advantageous solution for post-operative pain control.

The results we obtained align with these previous findings. Indeed, the use of ULF-TENS not only reduced pain but also caused an increase in voluntary mouth opening (MVO), which should not be underestimated. Patients with a reduced mandibular opening are, in fact, challenging for the dentist to treat optimally. Many of these patients, if required to keep their mouths open for an extended period, such as during an NSPT session, may experience difficult-to-manage and painful mandibular blockages. The use of TENS, by relaxing the masticatory muscles and acting on the temporomandibular joint, may reduce the frequency of these episodes [34,35].

However, our study has some limitations. As a pilot study, the sample size was intentionally small, limiting the ability to generalize the results. However, the choice of a crossover split-mouth design helped reduce inter-subject variability, thus improving the statistical power of the analysis. The small sample size inevitably influenced the age distribution of the patients included in the study, with a limited representation of individuals outside the 30–75 age range. For this reason, future studies should include a more heterogeneous population including pediatric and adolescent patients, as the use of TENS in younger populations could have a positive impact on anxiety reduction and improve patient cooperation during treatment. Altought all patients were treated by the same group of professionals; one limitation of our study is the lack of formal assessment of intra-examiner reliability. However, to minimize variability, all procedures followed a standardized protocol, and electrode placement was performed by an experienced operator specialized in TENS application for temporomandibular disorders. Future studies should include reliability assessments to further strengthen methodological rigor. Furthermore, a standardized protocol limited the ability to “blind” the treatment team. To improve the robustness of future results, studies could adopt a design where part of the team has no direct contact with the patients, allowing for more impartial data collection. Finally, we did not plan to compare TENS with the use of local anesthesia. Including local anesthetics as a study group would have added valuable insight to the findings. We acknowledge this limitation and intend to address this comparison in a future study with a larger sample size.

Despite these limitations, the results suggest that the use of ULF-TENS may represent a valuable tool for controlling pain and anxiety during non-surgical periodontal therapy. Its simplicity and relatively low cost make it a useful resource for clinicians in managing dental pain, with the potential to enhance the overall patient experience and treatment efficiency.

## 5. Conclusions

The findings from this pilot study suggest that Ultra-Low Frequency TENS (ULF-TENS) may be a useful adjunct for enhancing patient comfort during non-surgical periodontal therapy. Specifically, the use of ULF-TENS was associated with reduced pain and discomfort during the procedure, potentially improving patient satisfaction. However, these results should be interpreted with caution due to the limitations of the study. Further studies with larger, more diverse samples are needed to validate these findings and explore the broader applicability of ULF-TENS in clinical practice.

## Figures and Tables

**Table 1:** Population characteristics at baseline.

	1st Scenario Group (*n* = 10)	2nd Scenario Group (n = 10)
**Male (n)**	5	5
**Age (mean ± SD)**	46 ± 1.6	50 ± 0.5

## Data Availability

The data that support the findings of this study are available from the corresponding author upon reasonable request.

## Supplementary Materials

The following supporting information can be downloaded at: https://www.mdpi.com/article/10.3390/dj13040161/s1. Supplementary File Figure S1. Comfort level by age group with and without TENS. ANOVA test was performed to assess statistical differences, Supplementary File Figure S2. Comfort level within age groups with and without TENS. Paired-t test was performed to assess statistical differences, Supplementary File Figure S3. Maximum mouth opening pre and post TENS by age groups. ANOVA test was performed to assess statistical differences, Supplementary File Figure S4. Maximum mouth opening pre and post TENS within age groups. Paired-t test was performed to assess statistical differences, Supplementary File Figure S5. Pain level with and without TENS by age groups. ANOVA was performed to assess statistical differences, Supplementary File Figure S6. Pain level with and without TENS within age groups. Paired-t test was performed to assess statistical differences, Supplementary File Figure S7. VAS scale, Supplementary File Figure S8. DASS21 questionnaire.
